# Juvenile Hemochromatosis due to a Homozygous Variant in the *HJV* Gene

**DOI:** 10.1155/2022/7743748

**Published:** 2022-04-11

**Authors:** María-Belén Moreno-Risco, Manuel Méndez, María-Isabel Moreno-Carralero, Ana-María López-Moreno, José-Manuel Vagace-Valero, María-José Morán-Jiménez

**Affiliations:** ^1^Servicio de Hematología y Hemoterapia, Hospital Materno Infantil de Badajoz, Badajoz, Spain; ^2^Instituto de Investigación Sanitaria Hospital 12 de Octubre (imas12), Madrid, Spain; ^3^Servicio de Radiología, Hospital Universitario de Badajoz, Badajoz, Spain

## Abstract

Hemochromatosis type 2 or juvenile hemochromatosis has an early onset of severe iron overload resulting in organ manifestation such as liver fibrosis, cirrhosis, cardiomyopathy, arthropathy, hypogonadism, diabetes, osteopathic medicine, and thyroid abnormality, before age of 30. Juvenile hemochromatosis type 2a and 2b is an autosomal recessive disease caused by pathogenic variants in *HJV* and *HAMP* genes, respectively. We report a child with hepatic iron overload and family history of hemochromatosis. We aim to raise awareness of juvenile hemochromatosis, especially in families with a positive family history, as early diagnosis and treatment may prevent organ involvement and end-stage disease. The purpose of this study was to identify the gene variant that causes the disease. The genetic study was performed with a targeted gene panel: *HFE*, *HJV*, *HAMP*, *TFR2*, *SLC40A1*, *FTL*, and *FTH1*. We identified the variant c.309C > G (p.Phe103Leu) in the *HJV* gene in the homozygous state in the patient.

## 1. Introduction

Hereditary hemochromatosis (HH) is a disorder that causes excess iron absorption and its deposition in many organs. Progressive iron accumulation during decades result in illnesses such as liver fibrosis and cirrhosis, cardiomyopathy, diabetes, hypogonadism, hypothyroidism, arthropathy, and skin pigmentation [1]. Iron depletion by therapeutic phlebotomy or chelation therapy reduce morbidity and mortality [2].

The *HFE*, *HJV*, *HAMP*, *TFR2*, and *SLC40A1* genes have been identified as causative of HH types 1, 2a, 2b, 3, and 4, respectively. All of them have an autosomal recessive inheritance, except for type 4 with autosomal dominant inheritance [3, 4].

HH type 2a or juvenile hemochromatosis (OMIM 602390) is a severe type with an early onset, hypogonadism and cardiomyopathy being the most common symptoms at presentation and organ failure before age 30 [5, 6]. To date, more than one hundred cases of juvenile hemochromatosis have been reported with a prevalence of 1 in 5-6 million people worldwide [7, 8]. The hemojuvelin (*HJV*) gene is located in the chromosome 1q21.1, which encodes a protein involved in the iron metabolism. Hemojuvelin is a bone morphogenic protein coreceptor and acts as a modulator of hepcidin expression, a peptide that regulates the entry of iron into plasma [9]. Seventy-nine variants have been reported so far in the *HJV* gene (https://www.hgmd.cf.ac.uk/ac/index.php).

## 2. Case Report

**Figure 1:**
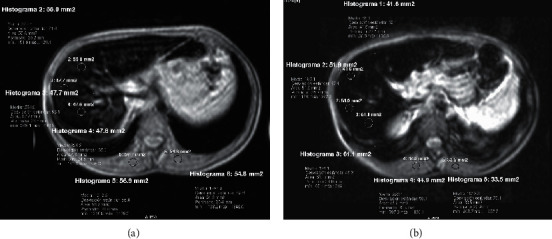
Abdominal MRI of patient. (a) Proton density fast field echo (PD FFE) sequence and (b) relaxation time T2 FFE sequence in the same axial section, with 3 regions of interest (ROI) in the liver and 2 ROIs in the paravertebral musculature to perform.

A child of Spanish origin with a familial history of iron overload and consanguineous parents was checked for ferritin, and he presented with hyperferritinemia since 3 years of age. Aged 8, he was diagnosed with hemochromatosis, presenting hyperferritinemia (777 ng/ml; normal range 30–400), high serum iron concentration (380 *μ*g/dl; normal range 50–120), and high saturation of transferrin (94%; normal range 20–50). Magnetic resonance imaging (MRI) of the abdomen was performed (Figure 1), and liver iron was quantified as indicated in https://www.sedia.es/calculadora-fe/, [10]. If the value is greater than 79 *μ*mol/g, it is compatible with high overload, and if it is greater than 85 *μ*mol/g, it is confirmatory of high overload. The patient showed a severe hepatic iron overload, 99.10 *μ*mol/g equivalent to 5.56 mg/g. Other liver tests were normal except for two punctual determinations of ALT/SGPT: abdominal ultrasound, no significant findings observed; ALT/SGPT, 26–46 U/l (normal range 9–39); AST/SGOT, 29–39 U/l (normal range 10–50); AF, 195–333 U/l (normal range 141–460); and GGT, 10–16 U/l (normal range 8–61). A study of Gilbert's disease due to intermittent hyperbilirubinemia (values 0.5–1.7 mg/dl; normal range 0.2–1.2 mg/dl) was recently requested, and the homozygous presence of the (TA)7/(TA)7 polymorphism compatible with Gilbert's syndrome has been detected in the patient.

Genomic DNA was extracted from a peripheral blood sample. The analytical method for genetic study was the Ion AmpliSeq™ Technology (Life Technologies, Carlsbad, CA, USA). A targeted gene panel was used to sequence coding, splice site regions, and 5ʹ and 3ʹ untranslated regions of genes *HFE*, *HJV*, *HAMP*, *TFR2*, *SLC40A1*, *FTL*, and *FTH1*, whose reference sequences are NM_000410.3, NM_213653.3, NM_021175.3, NM_003227.3, NM_014585.5, NM_000146.3y, and NM_002032.2, respectively. Torrent Suite 4.4 and Ion Reporter 5.2 softwares (Life Technologies) were used to analyze sequences and germline variants. Sequences were visualized in the Integrative Genomics Viewer (IGV-Human hg19). Sequences were reviewed and compared with the reference sequence by ClustalW2 software (https://www.ebi.ac.uk/Tools/msa/clustalw2/).

## 3. Discussion

The patient had the variant c.309C > G (p.Phe103Leu) in exon 3 of the *HJV* gene in homozygous state. These variants were inherited from his consanguineous parents. Moreover, this patient has the variant c.187C > G (p.His63Asp) in exon 2 of the *HFE* gene in heterozygous state. The variant c.309C > G in the *HJV* gene has been reported in a female patient in her thirties with iron overload in the liver and spleen [11].

**Figure 2:**
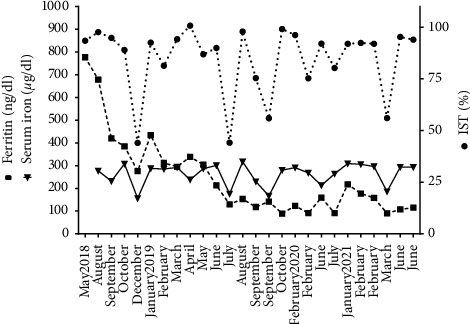
Serum ferritin and iron (left ordinate axis) and index saturation transferrin (right ordinate axis) of patient during phlebotomies treatment.

The hematologist prescribed phlebotomies of 7 ml blood/kg bodyweight weekly with the aim to achieving a serum ferritin concentration 50–100 ng/dl and a transferrin saturation index <50% [12]. The patient tolerated this therapy well though the process in such a small child was difficult, not only because of difficulty of venous access or peripheral catheterization dislodgement but also because the child was nervous and because the intercurrent infections altered the scheduled therapy. The therapy was suspended at the end of March and July 2019 due to infectious processes that required antibiotic treatment. From August 2018 to June 2021, the patient underwent thirty-four phlebotomies, and he achieved partial iron depletion (Figure 2). In the last MRI performed in May 2021, no hepatic iron overload was observed (19.4 *μ*mol/g equivalent to 1.1 mg/g). During this period, the hemoglobin remained in the range of 13–15 g/dl, the serum iron in the range of 156–317 *μ*g/dl, the serum ferritin in the range of 89–777 ng/ml and, the transferrin saturation in the range of 56–96%. Currently, the patient continues undergoing phlebotomies therapy. Chelation therapy is not considered at present.

A cardiologist, endocrinologist, and gastroenterologist are attending the patient who has had no organ manifestations such as cardiomyopathy, arthropathy, hypogonadism, osteopathic medicine, thyroid abnormality, glucose intolerance, or abnormal liver function test, but has skin pigmentation.

Phlebotomy is the mainstay of therapy for hemochromatosis to prevent iron overload [12]. This is particularly critical during childhood in the heart, pancreas, and pituitary gland that would lead to multiorgan dysfunction. Iron chelation is a treatment to reduce iron overload; this therapy has also been reported to reverse end-stage heart failure and to improve hematological and clinical parameters in patients with juvenile hemochromatosis [13–15].

Juvenile hemochromatosis is typically diagnosed during childhood, but a milder phenotype and late onset has been reported in some patients; therefore, genetic testing for variants in the *HJV* gene should be proposed in all cases of clinical expression of iron overload [11, 16–20].

A genetic study for the currently proposed genes responsible for hemochromatosis is recommended in patients with a phenotype of iron overload and in those patients with a positive family history. The earlier the diagnosis is made, the sooner treatment can be started to prevent complications derived from the progressive iron deposit in the body.
